# The Conformal Design of an Island-Bridge Structure on a Non-Developable Surface for Stretchable Electronics

**DOI:** 10.3390/mi9080392

**Published:** 2018-08-07

**Authors:** Lin Xiao, Chen Zhu, Wennan Xiong, YongAn Huang, Zhouping Yin

**Affiliations:** 1 State Key Laboratory of Digital Manufacturing Equipment and Technology, Huazhong University of Science and Technology, Wuhan 430074, China; linxiao@hust.edu.cn (L.X.); zhuchen@hust.edu.cn (C.Z.); xiongwn@foxmail.com (W.X.); yinzhp@hust.edu.cn (Z.Y.); 2 Flexible Electronics Research Center, Huazhong University of Science and Technology, Wuhan 430074, China

**Keywords:** island-bridge, conformal design, non-developable surface, stretchable electronics

## Abstract

Conformal design of the island-bridge structure is the key to construct high-performance inorganic stretchable electronics that can be conformally transferred to non-developable surfaces. Former studies in conformal problems of epidermal electronics are mainly focused on soft surfaces that can adapt to the deformation of the electronics, which are not suitable for applications in hard, non-developable surfaces because of their loose surface constraints. In this paper, the conformal design problem for the island-bridge structure on a hard, non-developable surface was studied, including the critical size for island and stiffness and the demand for stretchability for the bridge. Firstly, the conformal model for an island on a part of torus surface was established to determine the relationship between the maximum size of the island and the curvatures of the surface. By combining the principle of energy minimization and the limit of material failure, a critical non-dimensional width for conformability was given for the island as a function of its thickness and interfacial adhesion energy, and the ratio of two principal curvatures of the surface. Then, the dependency of the tensile stiffness of the bridge on its geometric parameters was studied by finite element analysis (FEA) to guide the deterministic assembly of the islands on the surface. Finally, the location-dependent demands for the stretchability of the bridges were given by geometric mapping. This work will provide a design rule for stretchable electronics that fully conforms to the non-developable surface.

## 1. Introduction

Stretchable electronics can be conformally transferred to various surfaces to perform multifunctional curvilinear electronics systems, such as electronic eye camera [1,2,3], 3D integumentary membranes [4,5], wearable devices [6,7,8,9,10,11], and smart aircraft skin [12,13]. The island-bridge structure is usually used in fabricating stretchable electronics, as it has made the most of high-performance, inorganic semiconductor materials. By placing intrinsic brittle materials on an unstretchable island to protect them from damage caused by strain, the whole device can suffer a large deformation without failure. When it is transferred to a hard, non-developable surface, strain will be produced in the device because of the geometric mismatch between the plane and non-developable surfaces, which may cause conformal problems for the device. On the one hand, although most of the strain is withstood by the bridge, strain still exists on the island. With the increase of the island size or the local curvatures of the surface, the strain on the island will increase as well and cause failure eventually. On the other hand, the strain in the device may cause the island to change position, which means stretchability is needed for the bridge to accommodate this change. Obviously, this demand for the stretchability of the bridges varies with the shape of the surface. Besides, the mismatch strain distribution is non-uniform, and it is dependent on the curvature distribution of the surface, which brings huge challenges in the deterministic assembly of the electronics. Considering that the island-bridge structure is a “mass-spring system” in the broad sense, the positon of the mass (island) in equilibrium can be decided once the stiffness of the spring (bridge) is known. So, it is possible to realize the deterministic assembly by predesigning the stiffness of the bridge. Hence, the conformal problems need to be studied to determine the critical size of the island, the demand for stretchability, and the stiffness of the bridge. 

The conformal problems of the island have been studied in epidermal electronics [14,15,16,17,18]. However, the target surfaces of epidermal electronics are usually soft and can accommodate the deformation of the island by being stretched or bent. Regarding conformal problem of island on a hard, non-developable surface, only the island is under deformation, which brings new challenges for the design of island. Several researchers have studied the adhesion and buckling problem between the elastic plate and the rigid sphere using theoretical, experimental, and simulation methods [19,20,21,22,23,24]. Majidi et al. [19] have given a critical conformal width for circular and rectangular elastic plates using the principle of energy minimization. However, the limits of material failure have not been taken into consideration, so it may not be suited to electronic design. Besides, the former studies are based on a sphere, which produces great limitations on the use of these theories. Mitchell et al. [25] show that a sheet that conforms to a cap and a saddle will produce different strain responses, respectively. Hence, a theory based on a more common surface needs to be proposed eagerly.

The theoretical works for the design of the bridge are quite mature, and many researchers have made significant contributions to this field [26,27,28,29,30,31,32,33,34,35]. Current works in bridge design mainly aim to promote its stretchability; the works for solving demand for stretchability are very rare. Nevertheless, some sacrifices are usually needed in other aspects of the device to obtain higher stretchability, such as functional duty ratio and material choice, which may cause an additional performance loss in the device. So, appropriate stretchability for the bridge is needed to be designed according to actual demand. On the other hand, the theoretical solutions for the stiffness of the bridges are mainly for thick bridges because of the complicated post-buckling behaviors in thin bridges [27,34]. Yihui Zhang [31] and Wentao Dong [32] have studied the thin bridge using finite element analysis (FEA), given its stretchability, but the relationships between stiffness and its geometric parameters for thin bridges are still ungiven. 

In the present study, the conformal behavior of the island and design demand for the bridge are studied. The layout of the paper is as follows. A mechanical model of the island on a part of torus surface is presented in Section 2, and a non-dimensional critical conformal width is given by the combination of the principle of energy minimization and the limits for material failure. Furthermore, an adhesion experiment for island is implemented to verify the validity of the theory. Section 3 describes the relationship between the tensile stiffness of the bridge and its geometric parameters by FEA. Furthermore, a location-dependent design strategy for the stretchability of bridges is given by geometric mapping. 

## 2. Conformal Criterion for Island

### 2.1. Conformal Modelling for Island

**Figure 1:**
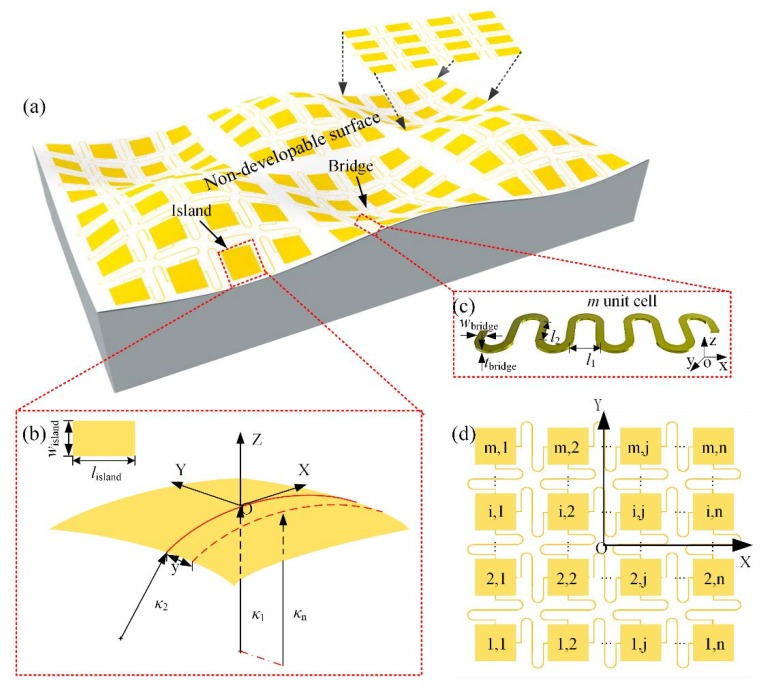
(**a**) An island-bridge structure array on a non-developable surface; (**b**) theory model of island on a torus surface under control by two principal curvatures, *κ*_1_ and *κ*_2_; (**c**) schematic of geometric parameters for a serpentine bridge with *m* unit cells; (**d**) a numbered island-bridge structure array with *m* rows and *n* columns of islands.

**Figure A1:**
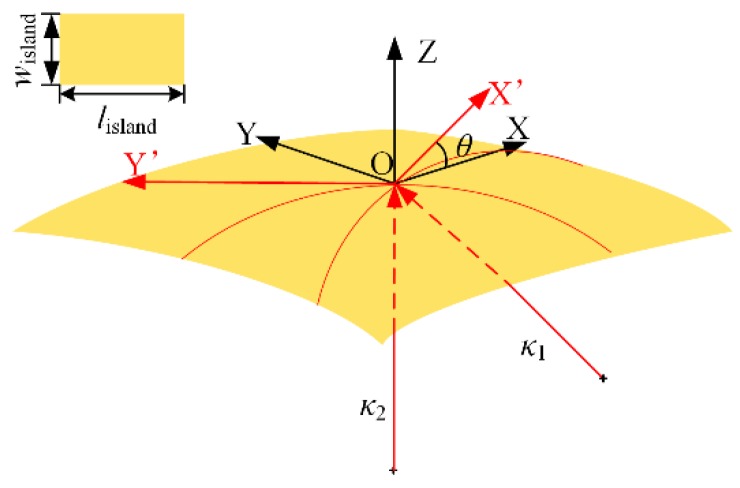
The schematic for island on surface with initial angle *θ*.

**Figure A2:**
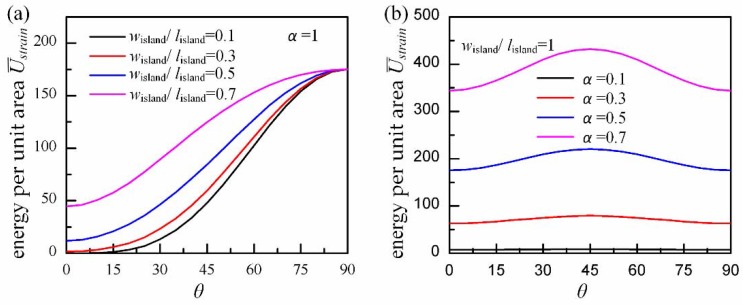
The conformal strain energy per unit area in island with initial angle *θ* at different width-length ratios (**a**) and different curvature ratios (**b**).

An island-bridge structure array is mapped onto a hard, non-developable surface, as shown in Figure 1a. The islands in the array are quite small compared to the target surface, so it is reasonable to use a small surface to approximate the local target surface covered by the island. Here, a torus surface under control by two principal curvatures, $\kappa_{1}$ and $\kappa_{2}$, is chosen for theoretical study. $\alpha \;=\;\kappa_{1}/\kappa_{2}$ is a geometric parameter that controls the shape of the surface. By appointing $\left|\kappa_{1}\right|\leq \left|\kappa_{2}\right|$, α is fixed among −1 and 1, which simplifies the analysis greatly. Different kinds of surfaces can be described by tuning α, such as saddle surfaces (for −1≤α<0), cylinders (for α = 0), paraboloids (for 0<α<1), and spheres (for α = 1). Then, an originally flat elastic island with length of *l*_island_, width of *w*_island_, and thickness of *t*_island_ ($t_{\mathrm{island}}\ll w_{\mathrm{island}}\leq l_{\mathrm{island}}$) is mapped onto a part of the torus surface under the assumption that no tension exists in width direction [19], which produces a rectangle conformal zone of length *l*_island_. Let the coordinates x and y denote the distance from the island center along the length and width direction, respectively, as shown in Figure 1b. Figure A1 shows the situation when the width direction is deviated from the bending direction of curvature $\kappa_{2}$ with a deflection angle *θ.* The relationship between conformal strain energy on the island and deflection angle *θ* is shown in Figure A2. It can be seen from the result that the island has lowest strain energy when *θ* = 0, which means a most steady state. So, we adopt this state to perform the analysis.

The strain in island for above problem is given as follows [36]:

(1) $$\begin{array}{l} \varepsilon_{xx}=\frac{\alpha \kappa_{2}z\cos\left(\kappa_{2}y\right)}{1+\left[\cos\left(\kappa_{2}y\right)-1\right]\alpha }+\left[\cos\left(\kappa_{2}y\right)-1\right]\alpha \\ \varepsilon_{yy}=\kappa_{2}z\\ \varepsilon_{xy}=0 \end{array}\;$$

**Figure A3:**
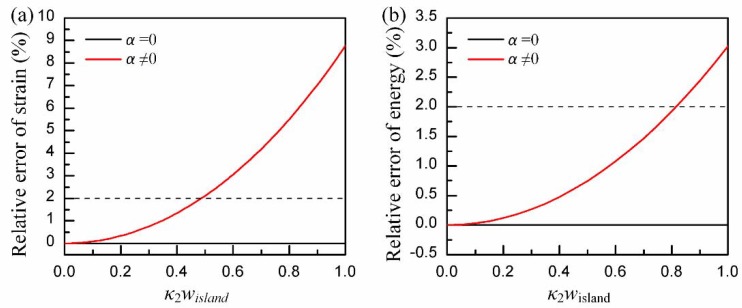
The relative error between approximate solution and accurate solution with $\kappa_{2}w_{\mathrm{island}}$: (**a**) for strain and (**b**) for conformal strain energy.

For the conciseness of energy integration, Equation (1) is replaced by an approximate one Equation (2) by Taylor expanding the $\cos\left(\kappa_{2}y\right)$. The error between Equation (1) and Equation (2) is below 2% when the non-dimensional width $\kappa_{2}w_{\mathrm{island}}\leq 0.5$, as shown in Figure A3a, which is reasonable for engineering application.

(2) $$\begin{array}{l} \varepsilon_{xx}=-\frac{1}{2}\alpha \kappa_{2}^{2}y^{2}+\alpha \kappa_{2}z\\ \varepsilon_{yy}=\kappa_{2}z\\ \varepsilon_{xy}=0 \end{array}\;$$

**Figure 2:**
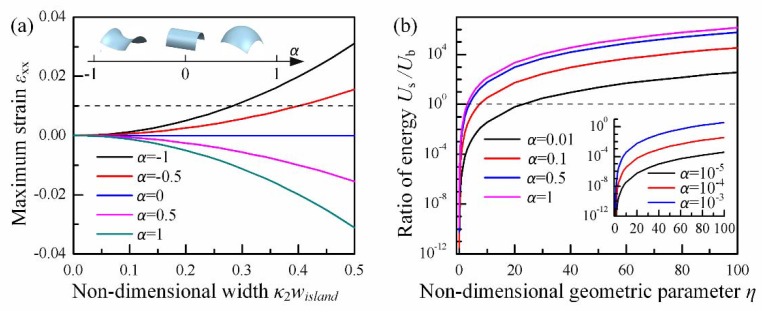
Strain and strain energy in the island during conformal contact: (**a**) maximum strain in island with non-dimensional width *κ*_2_*w*_island_ at *κ*_2_*t*_island_ = 10^−6^; (**b**) the ratio of stretching strain energy to bending strain energy with non-dimensional parameter *η*.

When α<0, the surfaces become saddles, and stretching strain will be produced in the island. With the increase of $\kappa_{2}w_{\mathrm{island}}$, the maximum strain in the island may exceed the failure strain 1% for most of inorganic materials on electronic applications, such as silicon [37] and zinc oxide [38], as shown in Figure 2a. When α>0, paraboloids are described; then, compressing strain shows up and may cause island buckling or failure. When the value of α decreases from 1 to 0, the surface tends to be a cylinder, and its developability is promoted, so the geometric mismatch strain in island is reduced. Specifically, when α = 0, the surfaces will turn into a cylinder upon which the island is under pure bending in y direction. 

By integrating Equation (2) in island domain, the strain energy on island is given below

(3) $$\begin{array}{ll} U_{strain} & =\frac{1}{2}{\bar{E}}_{\mathrm{island}}l_{\mathrm{island}}\int_{-w_{\mathrm{island}}/2}^{w_{\mathrm{island}}/2}\int_{-t_{\mathrm{island}}/2}^{t_{\mathrm{island}}/2}\left[\varepsilon_{xx}^{2}\left(y,z\right)+\varepsilon_{yy}^{2}\left(y,z\right)+2\nu \varepsilon_{xx}\left(t,z\right)\varepsilon_{yy}\left(y,z\right)\right]dydz\\ & =\frac{E_{\mathrm{island}}l_{\mathrm{island}}w_{\mathrm{island}}t_{island}^{3}\kappa_{2}^{2}\left(1+\alpha^{2}+2\nu \alpha \right)}{24\left(1-v_{island}^{2}\right)}+\frac{E_{\mathrm{island}}l_{\mathrm{island}}t_{island}w_{island}^{5}\kappa_{2}^{4}\alpha^{2}}{640\left(1-v_{island}^{2}\right)} \end{array}$$

where ${\bar{E}}_{\mathrm{island}}=E_{\mathrm{island}}/\left(1-\nu_{\mathrm{island}}^{2}\right)$, $E_{\mathrm{island}}$, and $\nu_{\mathrm{island}}$ are the Young modulus and Poisson’s ratio of the island, respectively.

Figure A3b shows an error of less than 2% when $\kappa_{2}w_{\mathrm{island}}\leq 0.8$ between the energy solution from Equation (3) and that from numerical integration of strain in Equation (1), which is acceptable for engineering application. The first item in Equation (3) is the energy contribution from bending (indicated as $U_{b}$) and the second one is from stretching (indicated as $U_{s}$). The ratio of energy contribution of those two deformations produces a non-dimensional geometric parameter $\eta =w_{\mathrm{island}}\sqrt{\kappa_{2}/t_{\mathrm{island}}}$. When *η* and *α* is small, the surface is nearly developable, so the bending energy is primary. With the increase of *η*, stretching behavior will contribute more to conformal energy and become dominant eventually, as shown in Figure 2b.

According to the principle of energy minimization, conformal contact is stable when $dU_{strain}/dw_{\mathrm{island}}\leq \gamma l_{\mathrm{island}}$, which implies

(4) $$\left|\kappa_{2}\right|w_{\mathrm{critical}1}=\sqrt[{4}]{\frac{128\left(1-v_{\mathrm{island}}^{2}\right)}{\alpha^{2}}\frac{\gamma }{E_{\mathrm{island}}t_{\mathrm{island}}}-\frac{16{\left(\left|\kappa_{2}\right|t_{\mathrm{island}}\right)}^{2}\left(1+\alpha^{2}+2\nu_{\mathrm{island}}\alpha \right)}{3\alpha^{2}}}\;$$

where $\left|\kappa_{2}\right|w_{\mathrm{critical}1}$ is the non-dimensional maximum critical conformal width from energy minimization and $\gamma /E_{\mathrm{island}}t_{\mathrm{island}}$ is the non-dimensional interface adhesion energy per unit area.

Meanwhile, the maximum strain in the island should not exceed the failure strain of functional materials on it, so that the electronics can keep working after being transferred to non-developable surface, which implies

(5) $$\varepsilon_{\max}=\left|\alpha \right|\left|\kappa_{2}\right|\frac{t_{\mathrm{island}}}{2}+\frac{1}{8}\left|\alpha \right|\kappa_{2}^{2}w_{\mathrm{island}}^{2}\leq \varepsilon_{\mathrm{critical}}\;$$

where $\varepsilon_{\mathrm{critical}}$ is the critical failure strain of functional material on island.

Hence, the maximum critical conformal width given by material limit is

(6) $$\left|\kappa_{2}\right|w_{\mathrm{critical}2}=\sqrt{\frac{8\varepsilon_{\mathrm{critical}}-4\left|\alpha \right|\left|\kappa_{2}\right|t_{\mathrm{island}}}{\left|\alpha \right|}}$$

By comparing two critical widths mentioned above, the final critical width for conformal is given by

(7) $$\left|\kappa_{2}\right|w_{\mathrm{critical}}=\min(\left|\kappa_{2}\right|w_{\mathrm{critical}1},\left|\kappa_{2}\right|w_{\mathrm{critical}2})$$

Referring to the curvilinear electronics system applications, they usually have mm-wide and μm-thick islands; a big enough *η* is almost satisfied, which implies that the stretching energy is dominant in conformal strain energy, so Equation (7) can be rewritten with

(8) $$\begin{array}{ll} \left|\kappa_{2}\right|w_{\mathrm{critical}}=\sqrt[{4}]{\frac{128\left(1-v_{\mathrm{island}}^{2}\right)}{\alpha^{2}}\frac{\gamma }{E_{\mathrm{island}}t_{\mathrm{island}}}} & when\;\xi \leq \xi_{\mathrm{critical}}\\ \left|\kappa_{2}\right|w_{\mathrm{critical}}=\sqrt{\frac{8\varepsilon_{\mathrm{critical}}}{\left|\alpha \right|}} & when\;\xi >\xi_{\mathrm{critical}} \end{array}$$

where $\xi =\gamma /\left(E_{\mathrm{island}}t_{\mathrm{island}}\varepsilon_{\mathrm{critical}}^{2}\right)$ is a non-dimensional parameter to comprehensively evaluate the effects of the adhesion energy and the failure strain of the material, and $\xi_{\mathrm{critical}}=1/\left[2\left(1-\nu_{\mathrm{island}}^{2}\right)\right]$ is constant given by making the above two critical widths equal (it depended only on the Poisson’s ratio of the island). 

**Figure 3:**
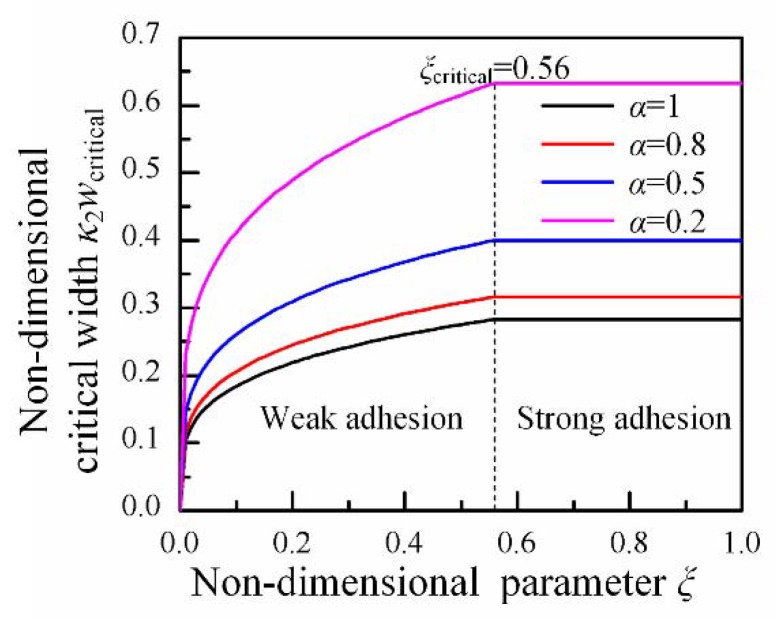
The non-dimensional critical conformal width $\kappa_{2}w_{\mathrm{critical}}$ with ξ for $\varepsilon_{\mathrm{critical}}=1\%$ and $\nu_{\mathrm{island}}=0.32$. Two regions, weak adhesion and strong adhesion, are divided by $\xi_{\mathrm{critical}}=0.56$.

The line $\xi \;=\;\xi_{\mathrm{critical}}$ divides the conformal domain into two regions, so-called ‘weak adhesion’ and ‘strong adhesion’, as shown in Figure 3. In the weak adhesion region ($\xi <\xi_{\mathrm{critical}}$), the critical conformal width increases with adhesion at the interface, which is consistent with the result given by Majidi [12]. Once $\xi >\xi_{\mathrm{critical}}$, it moves into the strong adhesion region. In this region, the critical conformal width is decided by material limit and will not increase with adhesion. The failure mechanisms of island in those two regions are quite different. In the week adhesion region, the maximum strain on the island remains below the failure strain during conformal contact, and detachment will occur at the interface when the adhesion is not able to afford to stable conformal contact. However, in the strong adhesion region, the adhesion is strong enough so that no detachment will happen. With the increase of the width of island, strain in the island will exceed the failure strain and cause the failure of the island eventually.

### 2.2. Adhesion Experiment for Island

**Figure 4:**
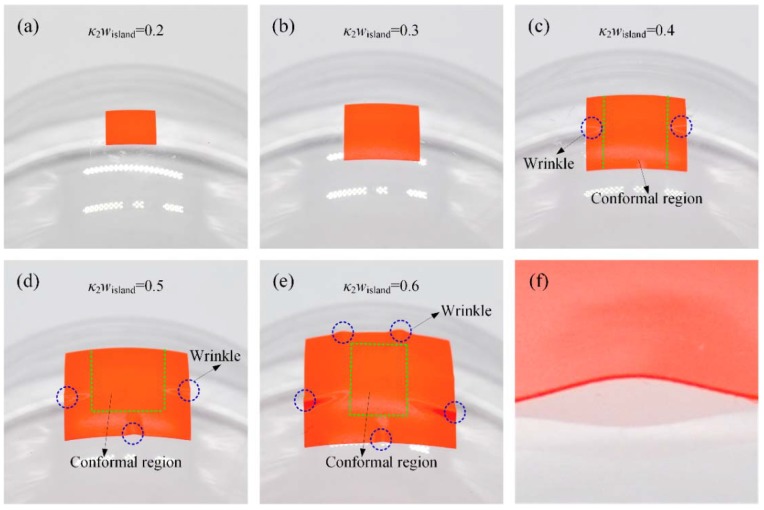
The conformal behaviors between sphere and PVC islands with different width: (**a**) $\kappa_{2}w_{\mathrm{critical}}=0.2$, (**b**) $\kappa_{2}w_{\mathrm{critical}}=0.3$, (**c**) $\kappa_{2}w_{\mathrm{critical}}=0.4$, (**d**) $\kappa_{2}w_{\mathrm{critical}}=0.5$, (**e**) $\kappa_{2}w_{\mathrm{critical}}=0.6$, and (**f**) enlarge view of wrinkle in (**c**).

Adhesion experiment is performed between polyvinyl chloride (PVC) sticker and Plexiglass sphere with a radius of 50 mm. The PVC sticker is carefully cut into a series of square islands with widths of 10, 15, 20, 25, and 30 mm ($\kappa_{2}w_{\mathrm{island}}=0.2,0.3,0.4,0.5,0.6$) by a cutting machine. Prior to the experiment, the spherical surfaces are scrubbed with alcohol and then air dried. Next, the PVC square island is slowly peeled off from the release substrate and pre-attached to the Plexiglass sphere to make sure that the center of the island is aligned with the sphere center. A soft stamp is used to apply pressure on the top of the PVC island to help further conformal contact. Here, a sponge is used as a stamp due to its negligible traction to the island. Finally, the stamp is removed slowly from the sphere, and the critical conformal width is measured after the conformal region remaining stable. When $\kappa_{2}w_{\mathrm{island}}<0.3$, the island completely conforms to the sphere, and no detachment is observed, as shown in Figure 4a,b. As $\kappa_{2}w_{\mathrm{island}}$ gets bigger, detachments will show up on both sides of the island (Figure 4c), and then the four sides of the island (Figure 4d,e). Due to the compressive strain in the island, the detached parts turn into buckling waves, as shown in Figure 4f. It is interesting to find that the bigger the width of the island is, the more buckling waves will be produced.

The thickness of the PVC sticker is measured by laser scanning confocal microscope (VK-X200, KEYENCE, Osaka, Japan), and a total thickness of 100 μm is given. Then, tension tests and peel tests are performed by a universal mechanical tester (INSTRON 5944, Instron, Norwood, MA, USA) and home-made peel platform to give Young’s modulus *E*_island_, Poisson’s ratio ν_island_, yield strain, and work of adhesion *γ*. The operational processes and test results for tension test and peel test are listed in Appendix C. For the adhesive PVC sticker used in the experiment, Young’s modulus, Poisson’s ratio, yield strain, and work of adhesion γ are $E_{\mathrm{island}}=1.29\;\;\mathrm{GPa}$, $\nu_{\mathrm{island}}=0.32$, $\varepsilon_{\mathrm{critical}}=2\%$, and γ=7.596  N/m, respectively. The non-dimensional parameter ξ for this experiment is 0.147, which is less than the critical one, which corresponds to a weak adhesion condition. The theoretical non-dimensional critical conformal width given by the first equation in Equation (8) is 0.2868, which is quite close to the experimental one ($\kappa_{2}w_{\mathrm{critical}}=0.3$). It is worth noting that with the further increase of the width of island after $\kappa_{2}w_{\mathrm{island}}>0.4$, the width of the conformal region will reduce (for $\kappa_{2}w_{\mathrm{island}}=0.5$, the conformal width is 0.26 and for $\kappa_{2}w_{\mathrm{island}}=0.6$, the conformal width is 0.24), which may come from the influence of the un-conformal region. As the width of island gets bigger, the un-conformal region gets bigger too, so the strain energy in the un-conformal region will be larger and larger. However, for the problem solving the critical width, this part of energy is not under consideration.

## 3. Mechanics of Stretchable Bridges

The design demands for bridges include two aspects. First of all, the tensile stiffness of the bridge needs to be designed so that the island can be deterministically assembled onto the target surface. On the other hand, the stretchability of the bridge needs to be designed to bear strain produced during the conformal process. In this section, the dependency of tensile stiffness of bridge on its geometric parameters and the demand for stretchability of the bridge on sphere are studied by FEA simulation and geometric mapping, respectively.

### 3.1. Tensile Stiffness Design for Bridges

A serpentine bridge with *m* unit cells is taken into consideration, as shown in Figure 1c. Each unit cell is composed of two half circles and two straight lines with length *l*_2_ and spacing *l*_1_ and has a rectangular cross section with width *w*_bridge_ and thickness *t*_bridge_. The serpentine bridge made of single layer PI with Young’s modulus $E_{\mathrm{PI}}=2.5\;\mathrm{GPa}$ and Poisson’s ratio $\nu_{\mathrm{PI}}=0.34$ is analyzed to given the scaling laws of axis force, and its dependency on the geometric parameters mentioned above. The tensile stiffness can be solved by taking a derivative of the axis force with respect to axial displacement. The serpentine bridge is clamped at two ends and pulls from an axial direction (x direction in Figure 1c). Four-node shell elements are used to model the serpentine bridge, and high-quality meshes are adopted to guarantee the accuracy of those analyses. A two-step method is used for FEA simulations. Firstly, the buckling analysis is adopted to get buckling strain and buckling modes for the serpentine bridge. Then, using the buckling modes from step 1 as initial imperfection to continue a nonlinear static analysis, a small enough damping is added to the model to ensure the convergence of the analysis.

**Figure 5:**
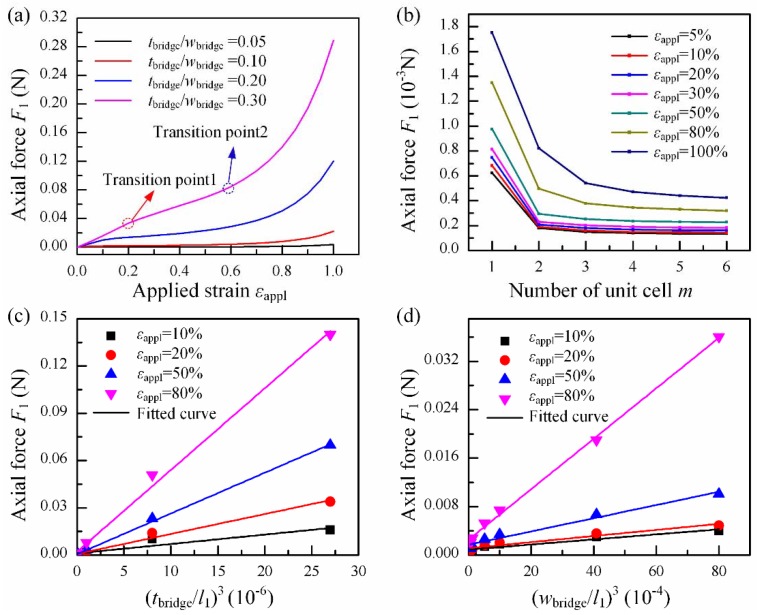
The axial force of a serpentine interconnect under stretching, obtained from the finite element analysis with different parameters: (**a**) applying strain, (**b**) wave numbers of bridge, (**c**) thickness of bridge, (**d**) width of bridge.

**Figure 6:**
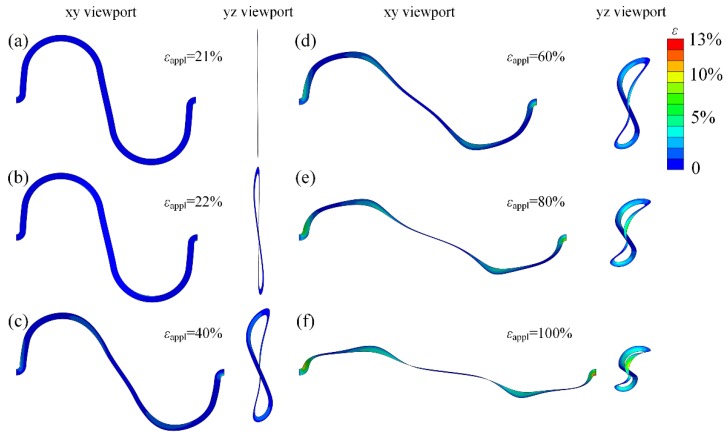
The max principal strain in the bridge versus the applied strain, and the corresponding deformation configurations in xy and yz viewport: (**a**) *ε*_appl_ = 21%, (**b**) *ε*_appl_ = 22%, (**c**) *ε*_appl_ = 40%, (**d**) *ε*_appl_ = 60%, (**e**) *ε*_appl_ = 80%, and (**f**) *ε*_appl_ = 100%.

The relationship between the axial force and apply strain reveals typical ‘J-shape’ stress-strain behavior, as shown in Figure 5a. The deformation of the serpentine bridge with strain shows a three stage, and two transition point are observed in simulations. The first stage is when the apply strain is lower than the critical strain for buckling. In this stage, only in-plane deformations exist, as shown in Figure 6a. The fact that the axial force keeps a linear relation with strain implies a constant tensile stiffness in this stage. The second stage starts with the buckling of the bridge when $\varepsilon_{\mathrm{appl}}\geq 22\%$, as illustrated in Figure 6b. In this stage, the bridge undergoes complicated bending and twisting deformation, and by comparing the configuration of the bridge in Figure 6c,d, it can be found that the tension between two ends is mainly matched by the rotation of the straight lines in the bridge. Hence, the tensile stiffness is in decline and maintains a constant approximately. With the increase of strain, it enters into the third stage. In this stage, the arc in the bridge begins to be straightened as shown in Figure 6e,f, so the tensile stiffness increases sharply with strain. 

Figure 5b shows the relationship between the axial force and the number of unit cells *m*. With the increase of *m*, axial force at the end of the bridge is decreased, which means a smaller tensile stiffness as well. Additionally, the effects of *m* tends to be saturated at *m* = 6. Figure 5c show a very good linear correlation between the axial force and the third power of the thickness of the bridge, and the same law can be seen in Figure 5d with the third power of the width, which corresponds to the contributions of out-plane and in-plane deformation, respectively. 

### 3.2. Stretchability Demands for Bridges

In this section, an *m × n* array of island-bridge structure with single island size *w*_island_ and distance *s* between two islands is mapped onto a sphere with radius *R*. The total length and width of the array are $l_{tot}=n\;w_{island}+\left(n-1\right)s$ and $w_{tot}=m\;w_{island}+\left(m-1\right)s$, respectively. 

For the island numbered as (*i*, *j*), its center coordinates are

(9) $$\begin{array}{l} X_{i,j}=\frac{2j-1}{2}\;w_{island}+\left(j-1\right)s-\frac{l_{tot}}{2}\\ Y_{i,j}=\frac{2i-1}{2}\;w_{island}+\left(i-1\right)s-\frac{w_{tot}}{2} \end{array}\;$$

Considering the unstretchable nature of the island, the demands for stretchablity of the vertical bridges during conformal processing can be given as follows by comparing the coordinates before and after the mapping

(10) $$\varepsilon_{i,j\to i+1,j}=\frac{1}{6}\frac{w_{\mathrm{island}}+s}{s}\frac{\left(Y_{i+1,j}^{2}+Y_{i+1,j}Y_{i,j}+Y_{i,j}^{2}\right)}{R^{2}-X_{i,j}^{2}}\;$$

where i,j→i+1,j means the bridge that connects two islands numbered as (*i*, *j*) and (*i* + 1, *j*) respectively.

In a similar way, the demands for stretchablity of the horizontal bridges are

(11) $$\varepsilon_{i,j\to i,j+1}=\frac{1}{6}\frac{w_{\mathrm{island}}+s}{s}\frac{\left(X_{i+1,j}^{2}+X_{i+1,j}X_{i,j}+X_{i,j}^{2}\right)}{R^{2}-Y_{i,j}^{2}}$$

**Figure 7:**
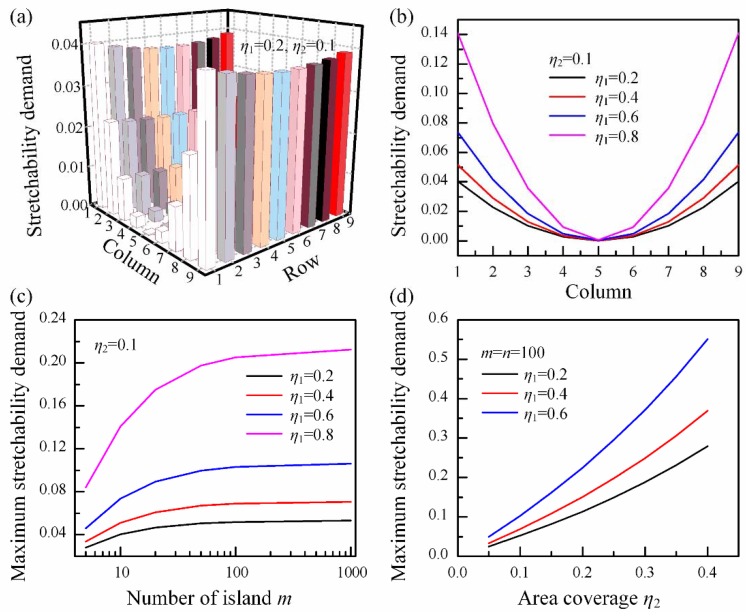
Demands for stretchability of the bridges given by geometric method: location-dependent property of demands for stretchability in the array (**a**) and at the first row (**b**) for the horizontal bridges; maximum demand for stretchability of the device with the number of islands (**c**) and area coverage (**d**).

Two parameters, functional coverage $\eta_{1}=w_{\mathrm{island}}/\left(w_{\mathrm{island}}+s\right)$ and area coverage $\eta_{2}=w_{tot}l_{tot}/\pi R$, are defined to describe the area ratio of sensor elements to the whole device and the device to the target surface, respectively. Figure 7 shows location-dependent demands for stretchability of horizontal bridges in the array. The same law is existent for vertical bridges as well. It is found that the bridges far away from the center of the device have higher demands for stretchability than those nearby. Hence, there are two design strategies for stretchability of bridge: one is using the maximum stretchability demand for all bridges in the array and another one is to design different stretchabilities for bridges at different locations. The former may be a convenient way, but as shown in Figure 7b, the demands for stretchability increase with $\eta_{1}$ sharply, and for $\eta_{1}=0.8$, there is 14% difference in numerical value between bridges at the edge and those near center, so this strategy will produce much redundancy in the whole device. However, a narrower wire width is usually needed for higher stretchability, which means a higher resistance as well. Hence, the latter strategy may be a more economical way. 

Figure 7c shows that the maximum stretchability demand increases with the number of islands in the array at the same functional coverage $\eta_{1}$ and area coverage $\eta_{2}$, and it tends to converge to a constant finally. For an array with larger $\eta_{1}$, such as $\eta_{1}=0.8$, the effect of the number of islands is more obvious, which implies that when we try to gain better conformability of the device by reducing the size of island, a higher stretchability will be needed. On the other hand, if the device is needed to cover a larger target surface with high functional coverage to obtain better performance, a higher stretchability will be needed, as shown in Figure 7d.

## 4. Conclusions

In this work, a theoretical model for the island conformed to a torus surface, governed by two principal curvatures $\kappa_{1}$ and $\kappa_{2}$ was set up. By adjusting the ratio of two principal curvatures, denoted as α, the conformal problem for island on saddle surface, cylinder, paraboloid, and sphere can be described. A non-dimensional critical conformal width was given for the island as a function of non-dimensional interfacial adhesion energy per unit area $\gamma /E_{\mathrm{island}}t_{\mathrm{island}}$ and non-dimensional thickness for the island $\kappa_{2}t_{\mathrm{island}}$ and α by combining the principle of energy minimization and the limit of material failure. A Poisson’s ratio relevant critical value $\xi_{\mathrm{critical}}$ divides the conformal domain into two regions, in which the adhesion and the limit of material failure are in charge, respectively. Besides, the relationships between the axial force of the bridge and its geometric parameters were revealed by FEA method so that the tensile stiffness of the bridge could be predesigned to help guide the deterministic assembly. Finally, a location-dependent demand for the stretchability of the bridge was found, and, based on this, an economical strategy was proposed by designing different stretchabilities for the bridge according to its location. Higher stretchability is a guarantee of better conformability. However, there are contradictions between stretchability and electrical performance; the collaborative optimization design is yet to be studied.

## Figures and Tables

**Figure A4:**
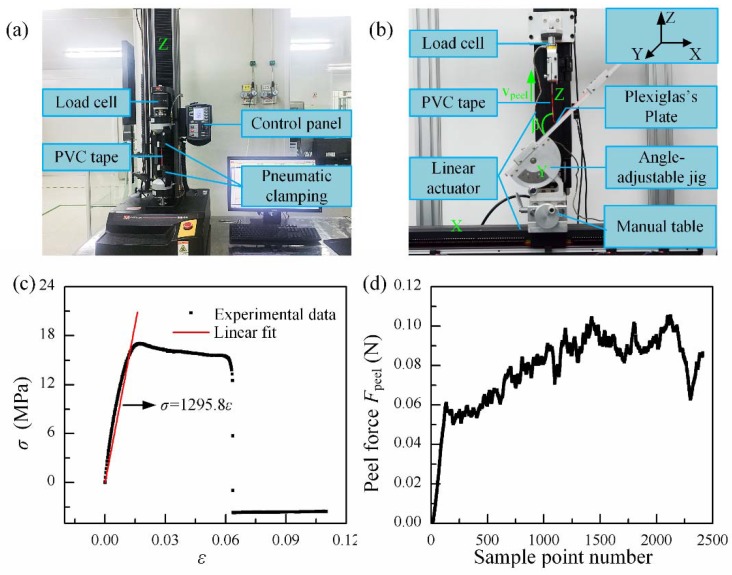
Experimental apparatus and experimental data for material parameter test: (**a**) an universal mechanical tester (INSTRON 5944); (**b**) a home-made peel platform with an angle-adjustable jig, in which the X or Z-motion of the translation stage is able to be driven by two independent linear/electric actuators, and its Y-motion depends on a manually single axis table; (**c**) stress-strain curve for PVC sticker in tension test; and (**d**) peel force for PVC sticker in peel test with peel angle $\beta =135^{\circ }$ and peel rate $v_{\mathrm{peel}}=1\;\mathrm{mm}/s$.

## Appendix A. Conformal Strain Energy with Angle of Deviation

Let coordinates OXYZ and OX’Y’Z, referring to island and surface, respectively, and the angle between them is marked as *θ*, as shown in Figure A1.

The strain components in coordinate OX’Y’Z are given by Equation (A1) as below

(A1) $$\begin{array}{l} \varepsilon_{x^{\prime }x^{\prime }}=\frac{\alpha \kappa_{2}z\cos\left(\kappa_{2}y^{\prime }\right)}{1+\left[\cos\left(\kappa_{2}y^{\prime }\right)-1\right]\alpha }+\left[\cos\left(\kappa_{2}y^{\prime }\right)-1\right]\alpha \\ \varepsilon_{y^{\prime }y^{\prime }}=\kappa_{2}z\\ \varepsilon_{x^{\prime }y^{\prime }}=0 \end{array}\;$$

where $\left[\begin{array}{c} x^{\prime }\\ y^{\prime }\\ z^{\prime } \end{array}\right]=T\left[\begin{array}{c} x\\ y\\ z \end{array}\right]$, $T=\left[\begin{array}{ccc} c & -s & 0\\ s & c & 0\\ 0 & 0 & 1 \end{array}\right]$ is the coordinate transformation matrix from coordinate OXYZ to coordinate OX’Y’Z, c=cosθ and s=sinθ.

The strain components in coordinate OXYZ can be solved from Equation (A1) by coordinate transformation

(A2) $$\left[\begin{array}{c} \varepsilon_{xx}\\ \varepsilon_{yy}\\ \varepsilon_{xy} \end{array}\right]=A^{-1}\left[\begin{array}{c} \varepsilon_{x^{\prime }x^{\prime }}\\ \varepsilon_{y^{\prime }y^{\prime }}\\ \varepsilon_{x^{\prime }y^{\prime }} \end{array}\right]\;$$

where $A=\left[\begin{array}{ccc} c^{2} & s^{2} & 2sc\\ s^{2} & c^{2} & -2sc\\ -sc & sc & c^{2}-s^{2} \end{array}\right]$ is the strain transformation matrix from coordinate OXYZ to coordinate OX’Y’Z.

Substituting Equation (A2) into Equation (3) and integrating the island domain using the numerical method, the strain energy on the island is given.

Figure A2 shows the relationship between conformal strain energy per unit area in the island and initial angle *θ*. When $w_{\mathrm{island}}/l_{\mathrm{island}}<1$, the minimum energy shows up when θ=0, which means that when the width direction is aligned with the bending direction of maximum curvature $\kappa_{2}$, the conformal status is most stable. This is why we have made this appointment in Section 2. When $w_{\mathrm{island}}/l_{\mathrm{island}}=1$, the minimum energy shows up in both $\theta =0^{\circ }$ and $\theta =90^{\circ }$; this is reasonable because of the symmetry of the square island.

## Appendix B. Relative Error between Approximate Solution and Exact Solution

To simplify the integral in Equation (3) and give a concise solution to critical conformal width, the strain in Equation (1) is approximated with Equation (2) using the Taylor expansion of $\cos\left(\kappa_{2}y\right)=1-\frac{1}{2}{\left(\kappa_{2}y\right)}^{2}+o\left({\left(\kappa_{2}y\right)}^{4}\right)$ when $\left|\kappa_{2}y\right|<1$. Figure A3 shows the relative error between approximate solution Equation (1) and accurate solution Equation (2). The accurate solution for strain energy is obtained by numerical integration from Equation (1). When α=0, the surface turns into a cylinder, and there is no error in the approximate solution. When α≠0, the relative error increases with non-dimensional width $\kappa_{2}w_{\mathrm{island}}$. Here, we choose 2% as the up limit of the error; thus, $\kappa_{2}w_{\mathrm{island}}\leq 0.5$.

## Appendix C. Material Parameter Test

The tension test is performed by a universal mechanical tester (INSTRON 5944, Instron, Norwood, MA, USA), as shown in Figure A4a. A dumbbell-shaped sample of PVC sticker with a length of 80 mm and a width of 5 mm in test section is connected to the tester by two pneumatic clampings. Then, the Z translation stage is moved upwards slightly to tighten the sample before the test. Finally, resetting the value of the load cell to zero and starting test with a tension rate of 1 mm/s. Figure A4c shows the stress-strain curve of the PVC sticker, and the slope of the curve, that is Young’s modulus, given by a linear fit of the elastic segment is 1.296 GPa. Another two samples are tested using the same method, and the Young’s modulae given by those tests are 1.298 GPa and 1.276 GPa, respectively. A mean value of 1.290 GPa is given as the Young’s modulus for PVC sticker. Figure A4c also shows a yield strain of approximate 2% for PVC sticker. Besides, the Poisson’s ratio is estimated from the percentage of cross-section contraction to its elongation.

Figure A4b shows a home-made peel platform whose peel angle and peel rate can be controlled. More detail about this platform can be found in [39]. A rectangle sample of PVC sticker with a length of 200 mm and a width of $w_{\mathrm{PVC}}=20\;\mathrm{mm}$ is prepared for test. Firstly, the sample is adhered to a Plexiglass plate, which is cleaned by alcohol and fixed at an angle-adjustable jig. Secondly, the sample is adjusted to the location right under the load cell by X and Y-motion, and the peel angle *β* is fixed at 135°. Thirdly, a part of the sample is peeled and its end is connected to the fixture of the load cell. After that, the location of the sample is adjusted again by X and Z-motion to make sure that the detached part of the sample remains upright. Finally, the value of the load cell is reset to zero and test is started with a peel rate of 1 mm/s. The force in the load cell starts at zero and keeps increasing before the beginning of the peeling at the interfaces, as shown in Figure A4d. The oscillation in force may come from the heterogeneous adhesion in PVC sticker or the motion of linear stepping motor; thus, the mean value of force in the peel segment is used as peel force. Three samples are tested to give an average peel force $F_{\mathrm{peel}}=0.089\;N$. Referring to the equation given in [39], the work of adhesion per unit area is $\gamma =F_{\mathrm{peel}}\left(1-\cos\beta \right)/w_{\mathrm{PVC}}=7.596\;N/m$.
